# Direct-to-consumer genetic testing: Prospective users’ attitudes toward information about ancestry and biological relationships

**DOI:** 10.1371/journal.pone.0260340

**Published:** 2021-11-29

**Authors:** James W. Hazel, Catherine Hammack-Aviran, Kathleen M. Brelsford, Bradley A. Malin, Laura M. Beskow, Ellen Wright Clayton

**Affiliations:** 1 Center for Biomedical Ethics and Society, Vanderbilt University Medical Center, Nashville, Tennessee, United States of America; 2 Center for Genetic Privacy and Identity in Community Settings (GetPreCiSe), Vanderbilt University Medical Center, Nashville, Tennessee, United States of America; 3 Department of Biomedical Informatics, Vanderbilt University Medical Center, Nashville, Tennessee, United States of America; 4 Department of Biostatistics, Vanderbilt University Medical Center, Nashville, Tennessee, United States of America; 5 Department of Electrical Engineering and Computer Science, Vanderbilt University, Nashville, Tennessee, United States of America; Universidad Internacional de La Rioja, SPAIN

## Abstract

Direct-to-consumer genetic testing is marketed as a tool to uncover ancestry and kin. Recent studies of actual and potential users have demonstrated that individuals’ responses to the use of these tests for these purposes are complex, with privacy, disruptive consequences, potential for misuse, and secondary use by law enforcement cited as potential concerns. We conducted six focus groups with a diverse sample of participants (n = 62) who were aware of but had not used direct-to-consumer genetic tests, in an effort to understand more about what people considering these tests think about the potential value, risks, and benefits of such testing, taking into account use by third parties, such as potential kin and law enforcement. Participants differed widely in the perceived value of direct-to-consumer genetic tests for ancestry and kinship information for their own lives, including the desirability of contact with previously unknown relatives. Some perceived ancestry testing as mere curiosity or entertainment, while others, particularly those who had gaps in their family history, few living relatives, or who were adopted, saw greater value. Concerns about intrusion into one’s life by purported kin and control of data were widespread, with many participants expressing concern about secondary uses of data that could harm users or their families. The use of direct-to-consumer genetic tests data for forensic genealogy elicited a particularly wide array of reactions, both spontaneously and in response to specific discussion prompts, mirroring the current public debate about law enforcement access to such data. The themes uncovered through our investigation warrant specific attention in the continued development of the science, policy, and practice of commercial direct-to-consumer genetic testing.

## Introduction

Many people want to know where they come from and to whom they are biologically related [1]. Family stories are handed down from generation to generation, and genealogy continues to be a popular activity, with countless books, websites, and television shows devoted to the subject [2]. Advances in genetics have added a powerful new tool to these inquiries, making it possible to obtain unprecedented insights into from where one’s forebears may have come (hereinafter, “ancestry”) as well as to whom they are biologically related (hereinafter, “kinship”), including discovering previously unknown relatives as well as contradicting understandings of established familial relations and lineages [3]. Many people incorporate information about these connections as part of their identity or how they see themselves in the world [4]. Yet evidence about origins and connections can raise concerns about privacy both for the individuals who undergo testing and for those with whom they are connected. People may want to limit access to information about them either on their own or by relying on a trustworthy system of governance, and much work demonstrates that many people worry that they will be harmed by downstream use of personal data [5]. Thus, searches for connections and desires for privacy can come into conflict.

Tens of millions of people have undergone direct-to-consumer genetic testing (DTC-GT) [6–8], many from companies that, until recently, marketed themselves as focused predominately on returning information about users’ ancestral origin and their potential biological relation to other people within the vendor’s database [9, 10]. Further, millions of these users have uploaded the genetic data underlying their DTC-GT results directly to independent websites to compare results with individuals who have undergone testing with other DTC-GT companies, often with the goal of identifying additional relatives [11, 12]. The search for kin is particularly common among adoptees and those who were knew they were conceived by gamete donation [13, 14]. Anecdotal reports describing the impact of genetic connections elucidated by the results of an individual’s own test, or those of a family member, have been widespread in the media since the earliest days of DTC-GT [15]. Some people are thrilled to find new relatives or uncover information about their ancestry [16], while others are distressed by these discoveries, particularly when they unearth family secrets [17] or when people they had not previously known reach out to them citing a genetic relationship [18].

Potentially complicating the public’s views about these tests, forensic genealogists have recently begun to use DTC-GT results shared by individuals to identify potential criminal suspects [19]. For example, law enforcement’s novel use of DTC-GT results from a publicly accessible genealogy database led to the identification of the Golden State Killer [20], solving a decades-long cold case. Use of these results by law enforcement, in some cases for uses beyond what the initial depositor intended, has not escaped the attention of DTC-GT companies, customers, or the public, whose responses range from applause to concerns about invasion of privacy [21, 22].

Although several studies have investigated public attitudes toward DTC-GT, these inquires tended to focus on health-related information, addressing ancestry and kinship only in passing [23–28]. A small number of studies have examined the reactions of people who have undergone DTC-GT and received information about ancestry or biological relatives [27–29], including a few focusing on the experience of adoptees [30]. But there remains a lack of data on perspectives and attitudes among the public more generally, their interest (or lack thereof) in such testing, and what people think these results would mean for themselves and their families [5, 31].

We sought in this project to investigate factors and rationales underlying expressed interest (or lack of interest) in undergoing DTC-GT for purposes of obtaining information about ancestry or identifying genetic connections with others. To this end, we explored what people who have not undergone these tests think specifically about the potential value, risks, and benefits of DTC-GT in the context of the distinct yet interrelated topics of ancestry, finding biological relatives, and forensic genealogy.

## Materials and methods

We conducted six focus groups with a diverse group of participants (n = 62) living in the greater Nashville, Tennessee, metro area, recruited via a professional research firm [32] (Complete participant demographics are found in Table 1).

**Table 1:** Participant demographics (n = 62).

Demographic	Response	*n*	%
Previous DTC-GT?	No	62	100%
	Yes	0	0.0%
Likelihood of Using DTC-GT to Learn More about Ancestry or Relatives *(pre-FGD)*	Very to Extremely Likely	15	24.2%
	Somewhat Likely to Likely	17	27.4%
	Neutral/Undecided	18	29.0%
	Somewhat Unlikely to Unlikely	8	12.9%
	Very to Extremely Unlikely	4	6.5%
Gender	Women	32	51.6%
	Men	30	48.4%
Age in years	21 to 29	3	4.8%
	30 to 39	11	17.7%
	40 to 49	16	25.8%
	50 to 59	14	22.6%
	60 to 69	9	14.5%
	70+	9	14.5%
Hispanic, Latinx, or Spanish Origin	No	59	95.2%
	Yes	3	4.8%
Race	White	49	79.0%
	African American, Black	11	17.7%
	American Indian	1	1.6%
	More than One Race	1	1.6%
	Other	0	0.0%
Education	Some High School	1	1.6%
	High School Graduate	8	12.9%
	Associate’s Degree	19	30.6%
	Bachelor’s Degree	19	30.6%
	Post Graduate	15	24.2%
Income	Less than $25,000	4	6.5%
	$25,000 to $49,999	16	25.8%
	$50,000 to $74,999	17	27.4%
	$75,000 to $99,999	12	19.4%
	$100,000 to $124,999	6	9.7%
	$125,000 or more	7	11.3%

Eligible participants were 1) at least 21 years old, 2) aware of but had *not* used DTC-GT, 3) had *not* participated in more than two medical or health-related research studies in the past year, and 4) did *not* work directly with genetics in their employment. As part of enrollment, participants rated their likelihood of using DTC-GT in the future to learn about biological ancestry and kinship. We assigned each individual to a specific focus group with the goal of maximizing diversity based on their self-reported demographics, as well as their initial rating of their likelihood to pursue testing, to represent a wide range of perspectives within each group. Focus groups were conducted only in English, although we *did not* exclude participants for whom English was not their first or primary language.

The Vanderbilt University Institutional Review Board deemed this research exempt under 45 C.F.R. §§46.104(d)(2)(ii). All focus groups were conducted in English in November 2019. The moderator (KMB) facilitated a group discussion in four sections: 1) DTC-GT *in general*, 2) *specifically for ancestry* information, 3) *specifically for kinship* information, and 4) *law enforcement access* to DTC-GT data. Notably, 2 and 3 addressed issues of identity, while 3 and 4 more directly confronted issues of privacy, as represented in Table 2 below.

**Table 2:** Organization and relevance of discussion topics and key content of corresponding educational components.

Topic	Key Education Content	Relevance to Identity and/or Privacy
**1. DTC-GT generally**	• introduction to at-home DNA test kits;	
	• procedure, process, cost;	
	• information returned to consumers (ancestry, kinship);	
	• common secondary uses of data (product development, commercialization).	
**2. DTC-GT specifically for *ancestry* information**	• defining ancestral origin, introduction to ancestry testing;	• Meaning and value of genetic ancestry in how one is perceived and understood by others and one’s self
	• how ancestry is approximated;	
	• accuracy and limitations of ancestry data;	
	• example of a typical ancestry report.	
**3. DTC-GT specifically for *kinship* information**	• defining kinship;	• Meaning and value of biological relation in how one is perceived and understood by others and one’s self
	• how relatives are identified using data generated for ancestry purposes;	
		• Role of biology in familial relationships
	• accuracy and limitations of kinship data;	
		• Potential contact with previously unknown relatives
	• example of typical kinship results;	
	• potential ways in which relatives may connect.	
**4. DTC-GT Data for Law Enforcement Use /Forensic Genealogy**	• overview of the use of ancestry/kin testing data for law enforcement purposes;	• Unanticipated and/or unapproved access to individual and familial information with direct identifiers
	• limitations of and constraints on use of DTC-GT data as supplement to traditional investigative techniques;	• Involvement and/or implication in criminal investigations
		• Extent to which engagement / contribution aligns with personal goals and values
	• example of forensic genetic genealogy;	
		• Choice and control over individual and familial information
	• consumer choice regarding law enforcement matching.	

**Fig 1:**
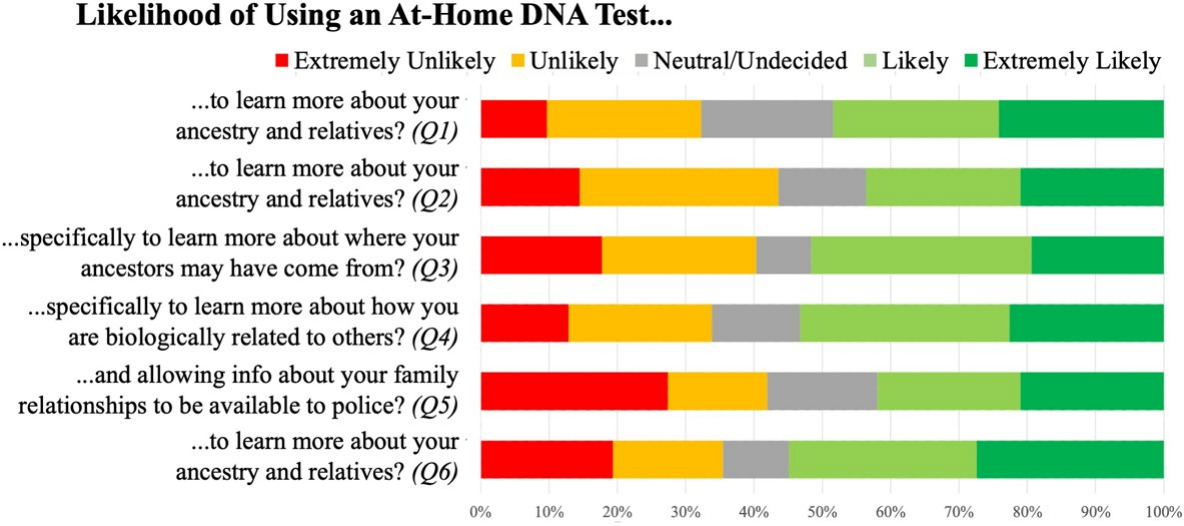
Participant worksheet responses. Questions correspond to the closed-ended questions asked throughout the focus groups (S3 Appendix). Q1 corresponds to the start of the focus group, prior to any educational materials.

A neutral expert in DTC-GT (JWH) introduced each section with an educational component comprising a standardized oral presentation with supplementary visual aids focusing on information essential for a basic understanding of the topic (*e*.*g*., how data are analyzed and results are determined as well as the type and validity/reliability of results), followed by an interactive question-and-answer period to resolve any confusion or misunderstanding (descriptions of the information provided to participants in the educational materials are found in Table 2). These educational components were specifically developed to establish a foundation for eliciting *informed* perspectives and opinions, rather than documenting misunderstandings. After each educational component, the moderator elicited participants’ perceptions and opinions regarding DTC-GT using a semi-structured guide (S2 Appendix). At key points, the moderator asked participants to respond to closed-ended questions on a worksheet (Fig 1 and S3 Appendix).

Groups lasted an average of 120 minutes, and participants received $100 for their time. All focus groups were audio recorded and professionally transcribed. We used an applied thematic approach to code, analyze, and interpret qualitative data [33]. Each transcript was reviewed by at least two members of the research team (CHA, EWC, JWH, LES, KMB, LMB) who independently identified potential codes capturing emergent thematic elements. Three team members (CHA, JWH, LES) then compiled, reviewed, and consolidated codes to construct an initial codebook. Next, they independently applied the codebook to one transcript, then met to compare code applications, resolve disagreements, and revise the codebook [34, 35]. This iterative process continued until the coders reached at least 80% agreement on all code applications. They then independently coded designated sections of each transcript, maintaining periodic intercoder agreement of at least 80% [33, 36]. Additionally, all coding was reviewed by one or more additional team member(s) for accuracy and completeness.

Saturation was monitored as FGDs progressed. After the completion of each FGDs, members from the research team in attendance discussed themes and opinions, identifying new perspectives with each additional group. Confirmation of saturation was more formally conducted during the coding process and demonstrated that >80% of all deductively and inductively coded themes were captured in the first three groups [37–39]. S1 Appendix provides additional details about the methods.

## Results

### The accuracy of direct-to-consumer genetic testing for defining ancestry and kinship

Most participants understood that DTC-GT could provide insights about ancestry and kinship, although some individuals in every group raised questions about the accuracy and stability of DTC-GT results, with some expressing worries about scientific errors. A few participants were surprised to learn that the accuracy of a user’s results depend on the pool of people who had previously been tested. Several opined that DTC-GT services are marketed as accurate and expressed surprise and disappointment that results were not necessarily complete or definitive. Specifically, kinship results would be limited by the extent to which a user’s biological relatives had also used DTC-GT and, if so, if they had used the same company (a topic briefly addressed in the educational materials, generating subsequent discussion in multiple focus groups), and ancestry results may have “*big gaps of emptiness*” (C03) or ancestry of unknown origins:

A05: I do want to fill in some gaps, but how do I know I’m filling accurate gaps with correct information?

### Meaning of ancestry and kinship information

#### Individual identity

Many viewed genomic data about ancestry and kinship as having a high importance or value to one’s identities:

C04: It’s more important. I don’t think it’s recreational as much as it is your own self-identity. […] it’s not just entertainment; it’s getting a better picture of who you are as a person and what kind of life story you’re going to leave behind.
--
F10: I guess because of the history of African Americans in the United States, it does have some bearing for me personally—we don’t know beyond my grandparents, really, where my family comes from. […] So, it would be nice just to have a sense of—and you hate to say—‘who you are,’ but there is a certain element of that in having that information.

Some perceived their ancestry to be particularly relevant to their individual identities but considered genetics to be less relevant to their ancestry than culture, tradition, and group inclusion. This concept that ‘ancestry’ comprised more than genetics was further elucidated when participants were asked to imagine receiving results that contradict their current beliefs about their own ancestral origin:

B01: I’m still Hispanic. […] If I find out that some great, great, great, whatever is from France—so? [I’m] Hispanic and still from Cuba, I’m not going to go say I’m from France. No, I’m Cuban and Dominican. Something that goes back 3,000 years ago? I’ll still be marking the same thing: Hispanic? Yes; White? Nope. That’s not going to change.

A few participants who indicated that genomic data about their ancestry and kinship could be relevant to their identities nonetheless expressed hesitancy or concern about what types of information DTC-GT data might reveal about their family histories, and how discovering “*something ugly*” (E04) might adversely affect their individual identities and perspectives:

A01: Me being African American, knowing what my ancestors went through and knowing that some slave owners raped some Black women. No, I don’t want to know if there’s a little bit of white—and it’s probably there—or European. I want to know my family’s history, but […] no, I don’t really want to know all that.
---
E04: What if you’re a product of rape? What if you’re a product of some real bad traumatic stuff? Which I really do think I am. I just wonder, what is the big thing that people are hiding? And what if it’s ugly and you’re better off never knowing it? Then someone’s like ‘Hey, I’m your cousin. Did you know that?’ and this wonderful rose-colored life that you’ve been living now turns into something [that you cannot unlearn].

Others viewed DTC-GT results as having little-to-no importance or value to them—with several echoing sentiments of “*I am who I am*” (D06)—contrasting ancestral origin information with other factors they perceived to be more influential to their identities and experiences:

F04: Nothing about my ethnicity defines who I am. The struggles I have in my own life—*those* define who I am.
--
D09: It would be mildly interesting to know it’s mostly Scandinavian or Italian or whatever. But for me personally, it doesn’t change my life one way or the other. I am who I am because of family I grew up in and the experiences that I’ve had. Where my ancestors came from doesn’t have an impact on my life today.

#### Impact of information on families

Beyond informing their personal understanding of their own roots, several participants specifically valued DTC-GT results—particularly relating to ancestry—as fostering a cohesive familial identity:

D03: There’s still a piece of me that I’m not familiar with. Knowing that I could share this information with my children, and then we could maybe travel to the place or learn more about this particular area or origin.

Most participants anticipated that discovering information about their ancestry and kinship—even if unexpected or incongruent with their family stories**—**would *not* have a substantial effect on their self-perception, individual or family identity, or established familial relationships. These participants emphasized that neither their personal identity nor their familial relationships are defined by biological connection; rather, they valued shared histories and memories, emotional bonds, and socially defined roles, standards, and expectations (*e*.*g*., loyalty) over biological connection:

C03: Relationships aren’t based or formed on DNA. They’re spiritual, they’re emotional, they’re historical.
C04: It wouldn’t make a difference to me. I might want to meet the milkman or the mailman, but I agree with pretty much everybody that it’s much more spiritual and what your relationship is than what your DNA says.
---
D03: I don’t think I would feel any different about them [if I learned we were not biologically related]. Especially if I’ve grown up with them my entire life, the love for them wouldn’t go nowhere. It wouldn’t faze me at all. Of course, there would be some questions like, ‘what’s going on?’ But as far as the relationship, it wouldn’t be anything different.

Several cited the value and stability of their relationships with non-biological family members:

A05: We have people that we claim as ‘family’ that are related by marriage. And people divorce, but they still come around to family gatherings, and they’re still just like family. So, [biological relation] doesn’t matter to me.
---
C03: Both of my daughters—one has married a young man that has a child and the other’s about to become engaged to a young man that has a child. Those are my grandchildren, without one drop of DNA.

While almost all participants ascribed to broader, social definitions of family regardless of biological relation, some nonetheless anticipated potential implications of receiving genomic data for one’s current family structure and dynamics. Many endorsed concerns that genomic data could expose unexpected or incongruent results unearthing family secrets, airing dirty laundry/finding skeletons in the family closet, or opening a “*can of worms*” (B04), which could be troublesome:

A05: I’m the type of person who lets sleeping dogs lie. I don’t want to dredge up drama between family members or anything like that. So, I would just leave it alone.
---
C05: I’d rather just live my life believing what I believe because, to me, it’s irrelevant at this point. What’s the point in causing yourself turmoil when you don’t have any? When people start looking for problems, this may be the way to find them.

Indeed, a few noted that familial disruption might arise not from uncovering unexpected connections *per se*, but from the revelation of dishonesty, concealment, or other actions by relatives violating trust or otherwise disrupting established familial relationships and narratives:

C05: If it was a sibling, I would look at my parents and have some questions, just because of so many years of not telling me. I think it could adversely affect that relationship because of the lie, not because of the biology.

Notably, several participants recounted their direct experience of learning unexpected information about their family trees, expanding hypothetical discussions into reality:

F06: I found out that my grandmother isn’t my biological grandmother a year ago. My mom thought that would break my heart, but it really didn’t because she raised me. So, if I found out like someone else in my family is that way, it really wouldn’t change anything for me because they are my family.

#### Connection and belonging

Many participants opined that DTC-GT information could be important to filling in gaps in one’s life story to foster connection and belonging:

D05: Everybody wants to belong to something. It’s just another extension of that, where you’re coming from and maybe what you have in common with other people you didn’t know you had before.
---
A09: I don’t have a lot of family. I’m here by myself. My grandparents have all passed. My parents are older with health issues. I would be excited to know that I wasn’t alone. I feel alone a lot.

Several participants noted missing or severed roots and branches in their family trees, and valued the potential for DTC-GT results to answer lingering questions:

B10: I tell everybody in my family every day, ‘I don’t belong to y’all. I know that.’ I know they’re not the family I belong to. […] I’m nothing like them. My dad is on every birth certificate of all six kids, except for one. I’ll let y’all figure out which one that was.

This participant went on to echo others who suggested that biological connection with a previously unknown relative could trigger certain expectations and obligations consistent with those of socially defined family:

B10: Blood relationship to me means […] you’ve got my back, and I’ve got your back, and we’re here for each other, and we do for each other, and we’re a family.

Although some participants suggested that DTC-GT results could foster new connections and affirm belonging, others suggested that new information—especially results that are unexpected or incongruent—may threaten established or potential connections with individuals and groups as well as individual identities based on such connections:

C05: My wife’s father is full-blooded Irish. [His town] has a big Irish St. Patty’s Day, and they march with the [*Irish surname*] banner. So, if they found out they weren’t Irish at all, I think it would make a big difference to them.

Among all groups, participants suggested that individuals who doubted or did not know their ancestry or their family’s biological relatedness or those with small families or whose relatives had died could find more value in DTC-GT for finding connection and belonging:

E06: I think if there were questions, if you had doubts your whole life because of half-truths or pieces just aren’t fitting together, it would be more intriguing. But if you look just like your parents or you have attributes like them, there’s not really much question there, so I wouldn’t feel like I would need to go have it validated. … But I think if you have already the lingering questions, or with adoption or wanting to find extended family, then [biological relation would be] really important.
---
F01: Let’s say I was raised and maybe I didn’t look anything like my family, and I had doubts. Maybe I heard whispers, maybe I heard rumors. I think something like that would be very valuable to a person like that. But someone like me—I know my family, my grandparents on both sides, there’s never been any doubts to parentage—I don’t think it would really matter. It would just be fun to find cousins because my family is small.

However, not all potential connections and memberships were viewed positively. Some were wary about the potential for genomic data to connect them to individuals or ideas with which they would not want to be associated, or “*tie you to something that you don’t have anything to do with”* (B04). Specifically, participants were concerned that biological relation to criminals (e.g., “*a serial killer*” (E06)) or other controversial individuals would tarnish their social reputation or public image:

B04: Pretend I get a testing done and I find out that my great-grandmother was Adolf Hitler’s sister, but she never told anybody because she was ashamed of that. Now I’m freaked out a little bit. … And then a couple of years later I want to run for [political office]. My opposition is going to say to their voters, ‘Do you really want somebody whose great-grandmother’s brother was Adolf Hitler?’
B08: They’ll just say, ‘related to Hitler.’
B04: That, to me, is that can of worms that people will just jump on. They jump on stuff that you didn’t even have anything to do with it.
B02: Somebody did something [many] years ago. You have no idea who they are, what they did—and it’s going to fall on you.

#### Weighing genetics against family narratives

While many participants expressed or implied high certainty of their own ancestral origins and biological familial connections, others highlighted the fallibility of family stories in general, noting that “*lots of people in this country have a very mixed background they don’t know that they have*” (E09):

A10: [There] is so much stuff that goes on in families. […] Rape, incest, teen pregnancies, and hiding stuff. And not really knowing the true story, you’d never know who you’re related to.
---
A06: Several centuries ago, females were not allowed to have kids and be unwed, and divorce was taboo. So, you made up a story to cover that part up because it can’t be known […] When your husband left you, you had to make up some story, ‘He died,’ instead of, ‘He ran off with my cousin.’ As it turns out, he didn’t die; he just skipped town because he didn’t want to be married no more. The story rolls downhill, generations after generations: ‘he died.’ But he’s living three counties over doing a whole different thing.

Several participants emphasized the value of genetic data to clarify/supplement, confirm, or correct one’s family story:

E07: [Oral history] is all by memory, it’s all by word of mouth, and this kind of tips the iceberg. […] The more data that’s out there, it gives you the opportunity to trace it yourself, and then you carry on that conversation, pass it down.

Regardless of their confidence in their own ancestry in particular, many participants found value in supplementing evidence such as genealogical records…:

F04: I would do it just to confirm what [my relatives] have researched out. My ancestry comes from Germany and Denmark and so I would just take it to see if it would correlate.

…and assumptions based on phenotypic traits, as illustrated by this Black participant while gesturing to their skin:

D02: I think in my family, I’m not certain but seems pretty apparent where they came from. But seeing that confirm or deny it would be interesting.

However, several participants reported that they would be *skeptical* of any results contradicting their established understandings/beliefs regarding their ancestry. These participants perceived genetic results to be *less* reliable than their family’s oral history, genealogical records, or other evidence such as phenotypic traits:

A08: I would question the validity of the test if it was something so completely opposite than what I had been told by family. […] If you had so much [genealogical] background from family bibles and that sort of thing, then something completely different would really make me question the test.

#### Contact with new relatives

When asked to imagine being contacted by previously unknown biological relatives, several participants expected that they would be skeptical of such individuals, citing the potential for scams, deceit, and misrepresentation/fabrication of results from imposters as well as the potential for ulterior motives in legitimate kin:

E08: In today’s age the way scams are, anybody can come by ‘Hey, I’m your cousin, I need a kidney.’ I’m just not comfortable with it.
E01: What’s their objective? What do they want? How much money you want to hit me up for? [There are] safety factors.

Some stated that they would *not* initiate or receive contact with new biological relatives. In addition to the aforementioned concerns about motive, these participants were wary of exposing family secrets, airing dirty family laundry/skeletons in the family closet, or otherwise disrupting existing familial dynamics:

B09: I wouldn’t want to impose. I don’t know what’s going on, what their family member told them. For me to open a can of worms, some hostile feelings. That’d be my reason for not reaching out to them. If they reach out to me, I’d be open, but I wouldn’t initiate. Not at all.

But others expected that they *would* initiate or receive contact with new relatives. For these participants, the risks were outweighed by anticipated benefits such as relieving loneliness, feeling connected to others, answering long-standing questions, clarifying the family story, and expanding their social support systems and networks:

B08: For me it would be the chance to not be alone. Most of my family are gone now, so if they’re willing to get to know me, it would be a chance to not be as alone as I am now.
---
E09: It would it be fascinating to find people like, "Oh my gosh, they actually look like me."
E03: Yeah. Or “we have the same laugh or the same eyes or the same fingers.” That’d be fascinating.

In discussing their potential willingness to connect with newly discovered kin, some participants stated that their level of interest and engagement would depend on contextual factors, such as the degree of relation:

E07: It would depend on how close of a relative it was. If it’s a second or third cousin, I mean, I have *first* cousins that I knew when I was a kid that I don’t talk to anymore, so I don’t need another one if it’s way down the line. If they’re really close, I’ll probably reach out.

Further, many expected that the extent of their communication would depend on specific characteristics of previously unknown biological relatives, emphasizing they would investigate any individual before communicating with them, expecting that new relatives would do the same for them:

D05: I would probably check social media first, just want to make sure they’re not way out there. I might know right then that we just aren’t going to get along or I’m probably not going to hang out with them, whether I’m blood or not.
D08: I’d like to know a lot more about them before I even tried.
D07: I would do a background check. And I would want to which aunt or uncle or grandparent they knew.
D09: Even if they may be related, you might want to know how much jail time they’ve done.

Some anticipated limited engagement or maintaining specific boundaries:

E02: I wouldn’t go and have dinner and drinks or something like that. But I might Facebook them and see about their life and let them see about mine, but no details.
---
A07: I would reach out to them, only on a level of if they were trying to research family. But as far as wanting to get together and be buddy-buddy, well … There’s enough people around me that I don’t need people that I don’t even have a clue about coming into my life right now.
A10: If I knew I had a distant relative, ‘let’s have some lunch, compare stories, information,’ and see if they’re weird or creepy, if they’re someone I want to continue back with or not for an actual interaction. But I wouldn’t stay at their house, though.

Notably, several participants recounted their personal experiences in contacting previously unknown relatives, illustrating the range of rationales, reactions, and effects:

E03: When I was adopted, those records were sealed. After my parents passed away, I pursued and got those records. … I made the call, and my purpose was I really wanted to just tell my birth parents thank you, because I was blessed with the most fabulous parents on the planet. I didn’t want to throw blame on anybody, I had no malicious intent. […] My birth father’s family totally embraced me. My birth mother, not so much—that door was just totally shut, and I am fine with that. But her family totally embraced me just like my birth father’s.

### The intersection of forensic genetics and ancestry/kinship testing

Participants spoke about the use of information from DTC-GT for forensic purposes, both spontaneously and in response to specific questions following an educational segment. Several participants shared personal anecdotes about law enforcement use of genetic data and recounted news or episodes in television, film, and literature, including several references to the high-profile Golden State Killer (GSK) case. Participants commonly asked questions about the accuracy of familial matches in the context of forensics, particularly for more distant relatives, the specifics of DTC-GT company practices with respect to law enforcement access (e.g., the process for opting in or out of law enforcement matching), and the law regarding compelled disclosure of data without respect to consumer choice (e.g., pursuant to a warrant or court order) and oversight (or lack thereof) of law enforcement more broadly. Law enforcement in the United States is typically able to engage in familial searches of the type used in the GSK case without a court order [40].

Following education about forensic genetics (S2 Appendix), we asked participants to brainstorm reasons why someone might *permit* use of their DTC-GT results for law enforcement matching. Many pointed to the utility of DNA in identifying and apprehending criminals, decreasing public safety threats, and reducing the cost, and increasing the efficiency of criminal investigations:

B07: I think of that Golden State Killer who was living next door to people, or the BTK killer who was continuing to commit crimes even though people had reported him. It would have been good had the daughter given DNA. […] They got someone who’s just done vicious killing that was living among people, [but] it could have shortened his reign of terror.

Additional considerations included the benefits of exonerating the innocent, locating missing persons, and bringing justice and closure to victims and survivors (“*Give peace of mind to those families that have* […] *never gotten the answer of where somebody is buried*, *where the person disappeared to*” (B07)). Overall, these discussions were often accompanied by altruistic statements about public service or a desire to contribute to the greater good:

A11: If it can help find someone, if it can help prosecute someone, if it can help exonerate someone, yeah, by all means.

We also asked participants to brainstorm why someone might *not permit* use of their DTC-GT results for law enforcement matching. A common theme was a general skepticism of law enforcement, including concerns about bad motives and misuse of the information…:

A08: Law enforcement, sometimes, aren’t the good guys. Maybe they’re using it for nefarious reasons unbeknownst to anybody else. I’m always hesitant or questioning this idea of ‘the greater good for everybody’ stuff.

…and/or mistrust of the government in general, with multiple participants referencing overreach, surveillance, “*Big Brother*” (A08), and other dystopian fiction:

C05: There’s just something that inherently bothers me about private information about me being out there. I read 1984 years ago, but now it’s really coming true because there are cameras everywhere. And the DNA—there’s something about it that inherently just bothers me.
---
D12: From all indications […] it seems this is something that the government is going toward whether we like it or not; they want to find out all they can about everybody. It’s just like Big Brother—they want to uncover everything they can, and it’s in the headlines daily [that] the governments want to do more and more to find out about the population.

One participant shared their insights as a former law enforcement professional:

A07: If you got a crime that happens out here, and you start with a pool of 750,000 people […] if you’ve got a DNA match, that shrinks that pool automatically. You can focus better, you got a better chance of *not* charging somebody [erroneously]. DNA is huge when it comes to helping exonerating people, and it’s huge when it comes to helping point somebody in the right direction. I knew officers whose investigative techniques were not good, and I’ve seen people railroaded in the past. Everybody has. But I’ve seen, also, officers do really good work and get the right folks most of the time. But yeah, it can be abused. Anything can be abused.

Participants also discussed the possibility that their DTC-GT results could implicate their biological relatives. Some expressed a desire to see criminals brought to justice, regardless of familial relationships, with a few specifying this as a key reason for permitting law enforcement access:

B07: We all have one family member we never trust. In every family, we have that one cousin, that one uncle, that one person […] you know he did something wrong. We just don’t talk about it.

In contrast, others cited the risk of exposing a relative to criminal liability as a reason to *not* permit law enforcement matching…:

A04: If you know your family has a less-than-beautiful reputation or a dicey history, that’s going to be a solid ‘no’ because you don’t want to hurt your tribe. They might not be great, but they’re still my people.

…or expressed more general reservations about exposing anyone to the criminal justice system, whether a close relative or not:

A10: I wouldn’t want a murderer to get off, but I wouldn’t want someone to misuse the information and affect someone’s life in a negative manner to where they can’t now take care of themselves or their family. Or they’re just thrown under the society’s bus. So, I’m torn.

Participants also discussed other factors that would enter into their decision, such as the nature of the crimes that were being investigated (often with the implication that the technique would be more permissible to investigate serious crimes, such as violence and crimes against children) and the level of oversight of forensic genealogical investigations. Others perceived that they had no choice about whether to permit law enforcement matching, expecting that law enforcement could access the information regardless of consumer preference.

Willingness to allow law enforcement to access DTC-GT results was the most polarizing topic covered in our focus groups, with participants nearly evenly split in their views: one-third reported they would be likely to extremely likely to share their results, one-third reported they would be unlikely to extremely unlikely to share, and the remainder was undecided/neutral [Fig 1, Q5]. At the conclusion of the focus groups, however, participants’ expressed interest in pursuing DTC-GT testing appeared largely unchanged from than their initial view [Fig 1, compare Q1 and Q6].

## Discussion

Genealogy is a major activity of millions of Americans, pursued to learn more about their own family history, which often informs their self-concept and their personal or familial identities [1, 2]. DTC-GT, by elucidating genetic relationships in the present and over millennia, adds powerful new tools for learning more about one’s ancestry and kinship. The use of these tests for this purpose, however, has received relatively little scholarly attention, particularly in comparison with research exploring views about health-related information from DTC-GT [41, 42]. In the early days of these tests, Wagner and Weiss (2012) reported that science and social bloggers writing about DTC-GT rarely discussed the potential implications of, or rationales for, pursuing such testing [43]. In a convenience survey conducted in 2010 of 176 people, these investigators in another article reported that primary rationales for pursuing DTC-GT were education and entertainment, while cost was the primary factor dissuading consideration. At that time, less than 20% of respondents were concerned about the validity of results or the policies and practices of DTC-GT companies and, on average, the respondents were ambivalent about any use by law enforcement. Other studies conducted during this period also addressed ancestry and kinship, albeit usually in passing [23–25].

Since that time, as the market in ancestry and kinship DTC-GT has exploded, interest in the attitudes of actual and potential users have evolved. For example, Yin et al. (2020) examined >150,000 comments posted on 23andMe and AncestryDNA subreddits (unsolicited by investigators) [44], finding that comments about ancestry and kinship dominated discussions, and results about biological relations with individuals elicited stronger and more divergent emotional responses than those about ancestral origins, paralleling those of our focus group participants. Ruhl et al. (2019) found that respondents to an online Mechanical Turk survey about DTC-GT (n = 1026), most of whom had not yet undergone testing, were more interested in ancestry than any other result and perceived ancestry and finding relatives as more likely to be informative than health-related results [22]. Horowitz et al. (2019) surveyed potential bone marrow donors, focusing specifically on ancestry testing [45]. While only about 5% of them had actually undergone DTC-GT, more than 95% said they would do so if it were free. The authors reported that White and Black respondents whose forebears had immigrated at least three generations earlier were more likely to be interested in having DTC-GT, while those who felt confident of their ancestry were less likely to be interested. Stallard and DeGroot (2020) conducted focus groups with self-identified family historians in several countries, reporting both how people engaged in genealogy incorporated DTC-GT into their practice as well as their views about potential threats to privacy and family disruption [28]. Saha et al. reported that actual and potential users of DTC-GT cited discovery of ancestry as a primary reason to pursue testing, while listing privacy, emotional toll, potential for misuse, and use by law enforcement as potential concerns [27].

The findings of our focus groups provide additional nuance to these recent studies. Many participants expressed concern about the accuracy and validity of DTC-GT results. These concerns were most prominent in the “initial reactions” phase of each focus group (prior to the educational component, which focused on the scientific basis and accuracy of these results), but they continued into the subsequent discussions of ancestry, kinship, and law enforcement.

They also expressed concerns, albeit to a lesser extent, about the accuracy of information regarding family relationships that could be derived from DTC-GT results. When asked to imagine that their test results contradicted family stories, many said they would discredit the DNA results more often than their family story. This is consistent with other studies’ findings that some people tend to believe a “truth” that aligns with their assumed and/or preferred ancestral background rather than DNA evidence leading to contrary conclusions [43, 45–51].

Participants also differed widely in the perceived value of DTC-GT for ancestry and kinship information for their own lives [47] As has been reported by others, a small number stated that they would not be tested so as to avoid findings that could alter their self-perception [29]. Many participants, however, viewed biological or genetic evidence of ancestral origin as unimportant or merely entertaining [43]. Similarly, in discussing what the results would mean for their views about kinship, many participants opined that lived experiences had much more meaning and importance than biological connections. This observation is consistent with the recent argument by Mathieson and Scally [52] that genetics is only one part of how people understand their ancestry, as well as more longstanding debates about the role of genetics in defining tribal membership [53, 54], group inclusion [26, 51], and other sociocultural structures from (and within) which people make meaning.

Our participants also varied widely in their views about the desirability of communicating with previously unknown relatives. As has been observed by others, some were eager to find kin, especially those who had few relatives or gaps in their family history [45] or who had been adopted [55]. Many, however, expressed concerns about intrusion on their personal privacy, being taken advantage of, and potential to upset existing family relationships and to create new obligations [28].

Participants’ concerns extended beyond the impact of how they would use the data themselves, worries that parallel broader debates about the extent to which people should be able to control secondary uses of data about them [5, 56]. Many were wary that data could be misused in a manner that could harm users and their families, echoing findings reported in other studies [22, 27]. Of particular note, while some participants supported the use of these data to find criminal suspects even among their own relatives, others were more cautious. This concern parallels the intense public discussion of such uses, fueled by a steady stream of media reports detailing successes associated with the widespread use of forensic genetic genealogy, the recent issuance of broad warrants targeting DTC-GT databases [57, 58], as well as the more general longstanding debate about appropriate limits on law enforcement’s use of genetic and other data [59]. In response to such hesitancy, some sites now offer users the opportunity to decide whether to permit use by law enforcement, while others advertise law enforcement access as a primary benefit of their services/platform [40].

Interpretation of our results is subject to several limitations. We have presented approximate proportions of participants who expressly addressed a topic, idea, or theme. These proportions are intended to indicate the extent to which themes were commonly discussed within and among groups; they are not intended to account definitively for each participant who may have supported, opposed, or generally discussed any given theme.

In addition, despite our efforts to maximize diversity across a range of demographic characteristics, our sample comprised somewhat fewer people of color than are represented in the population of Davidson County, where Nashville is located [60]. Due to the limitations of our sample and the qualitative nature of our study (even though this study is one of the largest qualitative examinations of these issues to date), it would be inappropriate to attempt to assess similarities and differences between and among demographic groups or to purport to reach conclusions about the public in general. Further investigation is needed to understand the extent to which perspectives and considerations differ between and among groups—particularly racial and ethnic minoritized groups.

Finally, we conducted these focus groups in October and November 2019 in the Greater Nashville Metro area. While our study was not intended to present the perspectives and considerations of a nationally representative sample, other studies suggest that the general themes and considerations presented here may well be broadly shared throughout the general public in the United States, though the prevalence of particular views may vary. This was also a period in which uptake of DTC-GT was beginning to wane [51–54], and before the disruption of the COVID-19 pandemic and the impact that the resulting isolation may have had on desire for connection.

## Conclusions

The dramatic uptake of DTC-GT offered for discovery of ancestry and kin has elicited a number of studies investigating actual and potential users’ opinions. This study adds to this literature by reporting the perspectives of individuals with no prior use of DTC-GT, and varying likelihood of future use, on the role of DTC-GT in ancestry and kinship specifically. While the value of DTC-GT is marketed and perceived as falling on a spectrum—from satisfying mere curiosity to revealing meaningful insights into one’s personhood and family tree—prospective consumers may also want to consider potential limitations, concerns about confidentiality and control of the data, as well as personal and familial implications. Discussion of the acceptability of using shared DTC-GT data for forensic genealogy was particularly prominent, eliciting a wide array of reactions, both spontaneously and in response to specific discussion, highlighting the salience of apprehensions about certain third-party applications. These themes and considerations warrant careful attention in the continued development of the science, policy, and practice of commercial DTC-GT.

## Supporting information

S1 Appendix Consolidated criteria for reporting qualitative studies (COREQ). (DOCX)

S2 Appendix Focus group moderator guide. (DOCX)

S3 Appendix Participant worksheet. (DOCX)

S1 Data (XLSX)
