# Supplementary material for: Direct-to-consumer genetic testing: Prospective users’ attitudes toward information about ancestry and biological relationships
Source: PLoS One. 2021 Nov 29;16(11):e0260340. doi: 10.1371/journal.pone.0260340 (PMC8629298; doi:10.1371/journal.pone.0260340)
Supplement: S1 Appendix — (DOCX) [file pone.0260340.s001.docx]

**S1 Appendix. Consolidated Criteria for Reporting Qualitative Studies (COREQ).**

| **Domain 1: Research Team and Reflexivity** | |
| --- | --- |
| **PERSONAL CHARACTERISTICS** |  |
| 1. Interviewer/facilitator: Which author/s conducted the focus groups? | All focus groups were conducted under the leadership of the Principal Investigator EWC (author). KMB (author) served as moderator. JWH (author) served as the neutral expert delivering all educational materials and responding to participant questions CHA (author) served as rapporteur. EWC and CHA directly observed all focus groups; LMB (author) observed one focus group. Research team members observing from an adjoining room were not visible to participants, but participants were informed of their presence |
| 2. Credentials: What were the researcher’s credentials? (*e.g. PhD, MD)* | James W. Hazel, PhD, JD; *Postdoctoral Research Fellow*; man; law, bioethics, direct-to-consumer genetic testing  Catherine Hammack-Aviran, MA, JD; *Associate in Health Policy*; woman; law, bioethics, qualitative research  Kathleen Brelsford, PhD, MPH; *Social Scientist*; woman; qualitative research, methodology, medical anthropology  Laura M. Beskow, PhD, MPH; *Professor of Health Policy*; woman; health policy, research ethics  Bradley A. Malin, PhD, *Professor of Biomedical Informatics;* man, computer science, privacy, genomics  Ellen W. Clayton, JD, MD; *Principal Investigator*; *Professor of Pediatrics, Law, and Health Policy*; woman; law, bioethics, genomics  Lisa Smilan, JD, LLM; *Postdoctoral Research Fellow*; woman; law, ethics (acknowledged) |
| 3. Occupation: What was their occupation at the time of the study? |  |
| 4. Gender: What is the researcher’s gender? |  |
| 5. Experience and training: What experience or training did the researcher have? | Each author has at least ten years of research experience in qualitative and/or quantitative methodologies (including the conduct of group interviews and qualitative coding and analysis). |
| **RELATIONSHIP WITH PARTICIPANTS** |  |
| 6. Relationship established: Was a relationship established prior to study commencement? | No relationship was established between the moderator/expert and participants prior to study commencement. To our knowledge, no participant had a previously established relationship with another participant. |
| 7. Participant knowledge of the interviewer: What did the participants know about the researcher? *(e.g., personal goals, reasons for doing the research)* | Participants were informed that the moderator/facilitator (KMB) and neutral expert (JWH) were researchers from Vanderbilt, were not affiliated with any DTC-GT commercial company, and that the goal of the focus group was to solicit opinions and perspectives surrounding “at-home DNA test kits.” |
| 8. Interviewer characteristics: What characteristics were reported about the interviewer/facilitator? *(e.g., bias, assumptions, reasons and interests in the research topic)* | At the start of the focus groups, participants were informed that the moderator/facilitator (KMB) and neutral expert (JWH) were unbiased (i.e., had a neutral opinion of DTC-GT and were interested in soliciting a broad array of opinions and perspectives) and had no affiliation with a DTC-GT company. |
| **Domain 2: Study Design** |  |
| **THEORETICAL FRAMEWORK** |  |
| 9. Methodological orientation and Theory: What methodological orientation was stated to underpin the study? *(e.g., grounded theory, discourse analysis, ethnography, phenomenology, content analysis)* | We used an over-arching grounded theory research methodology. Within the overall framework, we employed an applied thematic analysis (including constant comparative analysis) to identify and refine meaningful categories.  LMB was instrumental to defining the research questions, the focus group guide, the worksheet, and the educational components, as well as the IRB application and contract with 20\|20 Research, Inc., working closely with KMB, CHA, and JWH, and in collaboration with EWC. |
| **PARTICIPANT SELECTION** |  |
| 10. Sampling: How were participants selected? *(e.g., purposive, convenience, consecutive, snowball)* | As described under *Materials and Methods,* 20\|20 Research, Inc., was contracted to recruit a sample of participants who reported that they were: ≥21 years old; aware of but had *not* used DTC-GT; had *not* participated in more than two medical or health-related research studies in the past year; and did *not* work directly with genetics in their employment. |
| 11. Method of approach: How were participants approached? *(e.g. face-to-face, telephone, mail, email)* | As described under *Materials and Methods*, participants were recruited by 20\|20 Research, Inc., a professional research recruitment firm. |
| 12. Sample size: How many participants were in the study? | n = 62 |
| 13. Non-participation: How many people refused to participate or dropped out? Reasons? | Of the 63 individuals who were recruited by 20\|20 Research, Inc. and arrived to focus groups, one (1) individual withdrew from a focus group in progress due to illness; that participant’s data is excluded. |
| **SETTING** |  |
| 14. Setting of data collection: Where was the data collected? *(*e*.g., home, clinic, workplace)* | Data was collected at the 20\|20 Research, Inc. facility in Nashville, TN. |
| 15. Presence of non-participants: Was anyone else present besides the participants and researchers? | No. However, some research team members observed the focus groups from an adjoining room (though not visible to participants). Participants were informed of their presence. |
| 16. Description of sample: What are the important characteristics of the sample? *(e.g., demographic data, date)* | The sample is described in detail under *Materials and* *Methods: Participants* and under *Results: Participant Characteristics* (Table 1). |
| **DATA COLLECTION** |  |
| 17. Interview guide: Were questions, prompts, guides provided by the authors? Was it pilot tested? | The group interview questions and prompts associated with the data reported here are described throughout the text and in Fig 1; a summary of the educational components are also provided (Table 2). The instrument and materials are available upon request. |
| 18. Repeat interviews: Were repeat interviews carried out? If yes, how many? | No data collection was repeated. |
| 19. Audio/visual recording: Did the research use audio or visual recording to collect the data? | With each participant’s permission, all focus groups were digitally audio-recorded. |
| 20. Field notes: Were field notes made during and/or after the interview or focus group? | Yes; researchers took notes throughout the focus group discussions, as well as additional post-discussion contextual notes when relevant. The rapporteur (CHA) verbally narrated non-verbal behaviors (e.g., body language, facial expressions) on a separate audio recording aligned with the discussions. |
| 21. Duration: What was the duration of the interviews or focus group? | Each focus group discussion lasted approximately 120 minutes. |
| 22. Data saturation: Was data saturation discussed? | After coding all transcripts, no additional themes were identified to add to the codebook, suggesting saturation. |
| 23. Transcripts returned: Were transcripts returned to participants for comment and/or correction? | No |
| **Domain 3: Analysis and Findings** |  |
| **DATA ANALYSIS** |  |
| 24. Number of data coders: How many data coders coded the data? | Three (3; CHA, JWH, LS) |
| 25. Description of the coding tree: Did authors provide a description of the coding tree? | The systematic application of structural and content codes is described under *Methods*. The headings and subheadings used in the manuscript reflect the basic structure of the coding tree. |
| 26. Derivation of themes: Were themes identified in advance or derived from the data? | Themes were derived from the data. |
| 27. Software: What software, if applicable, was used to manage the data? | NVivo 12 (2018) |
| 28. Participant checking: Did participants provide feedback on the findings? | No |
| **REPORTING** |  |
| 29. Quotations presented: Were participant quotations presented to illustrate the themes / findings? Was each quotation identified? *(e.g., participant number)* | Participant quotations are presented, and each quote is identified by participant number. |
| 30. Data and findings consistent: Was there consistency between the data presented and the findings? | Our manuscript integrates extensive use of direct quotes to provide evidence for each conclusion drawn. |
| 31. Clarity of major themes: Were major themes clearly presented in the findings? | Major themes are clearly identified within distinct headings and subheadings. |
| 32. Clarity of minor themes: Is there a description of diverse cases or discussion of minor themes? | There is substantial discussion of themes within each subheading, including diverse cases and minority opinions. |
