# Supplementary material for: Direct-to-consumer genetic testing: Prospective users’ attitudes toward information about ancestry and biological relationships
Source: PLoS One. 2021 Nov 29;16(11):e0260340. doi: 10.1371/journal.pone.0260340 (PMC8629298; doi:10.1371/journal.pone.0260340)
Supplement: S2 Appendix — (DOCX) [file pone.0260340.s002.docx]

**S2 Appendix. Focus Group Discussion (FGD) Moderator Guide**

| **INITIAL REACTIONS & BASIC INFORMATION** |
| --- |

For the next few hours we are going to talk about at-home DNA testing kits, where you can send a saliva or spit sample to a company and they will send you information about what parts of the world your ancestors may have come from and information about how you are biologically related to others. We are *not* talking about genetic testing that you might have heard of or had done to learn more about your health.

We have invited an expert on this topic to provide us with some basic information about these types of kits. [JH] is a lawyer and has training in genetics. He’s also spent several years learning about different testing kits and how they work. At various points today, he will be telling us a little bit about testing kits and can answer questions you might have about them.

But before hearing from our expert, I’d like to hear some of your initial thoughts about these kinds of testing kits – and again, just to remind you, we are talking about kits that tell you where your ancestors might have come from and who you might be related to. We are *not* talking about health-related testing.

1. To begin, what you have heard about these kinds of testing kits?
2.
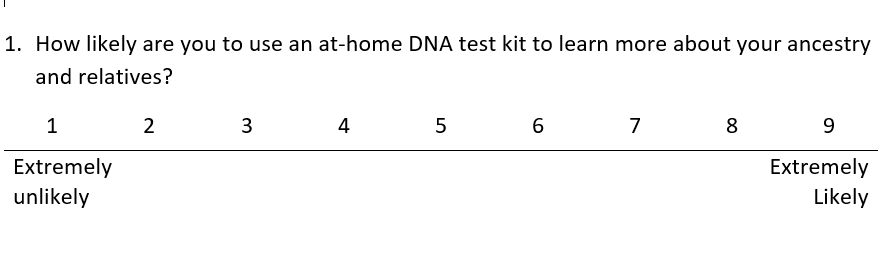
 Please turn to Question #1 on your worksheet. Please circle the number from 1-9 that best reflects your likelihood of having this type of testing done in the future, where 1 is “extremely unlikely” and 9 is “extremely likely”. If you are having trouble choosing between two numbers on the scale, please choose the one that better reflects how you’re currently feeling.

3. I’d like to briefly hear some of the reasons you are or aren’t likely to have testing done in the future.

a. For those of you who rated your likelihood between 1 and 3 on the scale, briefly tell us why you’re *unlikely* to have it done.

b. For those of you who rated your likelihood from 7 to 9, what are some of the reasons you’re *likely* to have testing done?

c. For those of you who had ratings between 4-6, what are some of the reasons you feel neutral or undecided?

| **Educational Component: DTC-GT, general** |
| --- |
| **What Is At-Home DNA Testing?**   - At-Home DNA testing looks at differences in a person’s DNA in order to provide them with information. People may do this testing for a variety of reasons - Today we will focus on what at-home DNA testing can tell you about:   1. Your Ancestry   2. Your Relatives and Family Tree   **At-Home DNA Testing: How It Works**   1. Purchase Test Kit (online or in store) 2. Provide Sample (saliva or cheek swab) 3. Mail Kit Back (for DNA testing) 4. Receive Results (online or by mail)  - The entire process takes place in your home without the help of a doctor to order the test or receive the results.   **How Companies Use Your DNA and Information**   - In addition to providing information about your ancestry or relatives, companies may also: - Use the information to develop new products or improve the quality/accuracy of their existing products   - Sell the information to researchers and scientists     - For example: universities and medical centers   - Sell the information to other companies     - For example: pharmaceutical companies   *Key Points:*   - What ancestry/kin testing is - Procedure, process, cost - What you get (testing can indicate ancestry and kin relations) - Uses (basic research, marketing, personal information) |

Q4. Is there anything new or surprising that you didn’t know before?

Q5. Now that you know a bit more about this kind of at-home testing kit, I’d like you to again rate your likelihood of having it done.


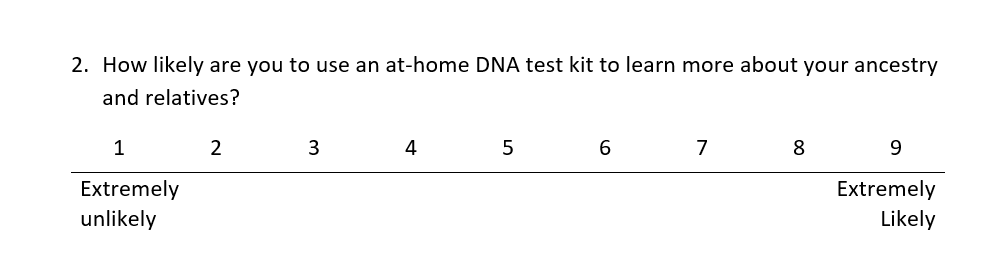


Compare this rating to your rating for Q1.

- 1. How many of you had a different rating? For those of you who had a different rating, how many of you are now *more* likely to have testing done? Talk to us about why.

- For those of you who are *less* likely to have testing done, talk to us about why.

- 1. For those of you whose reasons did not change, did any of you have new or different reasons that you considered?

| **ANCESTRY INFORMATION** |
| --- |
| **Educational Component: DTC-GT for Ancestry Information** |
| **Ancestry Testing: How it Works**   - By comparing your DNA with the DNA of other people from around the world, **a** DNA test can tell you where people who have DNA that is similar to yours can be found on the Earth today. - Ancestry testing is not 100% accurate   - Ancestry tests are more accurate for people of certain ancestries than others   - Different companies may offer different estimates of ancestry   - Ancestry estimates may change over time   **Example of an Ancestry Report**  [*A stock image shows ‘Jane,’ a racially/ethnically ambiguous woman in young-to-mid adulthood. To her right is an example of an ancestry report reflecting Jane’s ancestral origins in Great Britain, Asia, and Congo/Cameroon at roughly equal proportions (~22-35%) alongside Asia (2%) and “Other” (17%).*]  *Key Points:*   - How ancestry is approximated - The information a consumer receives regarding ancestry |

Q6. Is there anything new or surprising that you didn’t know before?

Q7. What meaning—if any—would it have to you to know from which parts of the world your DNA might have come from? In other words, how much does it matter to you to know where your ancestors came from?

Q8. Regardless of how certain you might be of your ancestry, let’s image you got a result that was different from what you expected. How would you feel? What would be your reaction?

Q9. Now that you’ve had a chance to think through what different kinds of results might mean, how likely would you be to pursue testing *specifically to learn more about* ***where your ancestors came from***?


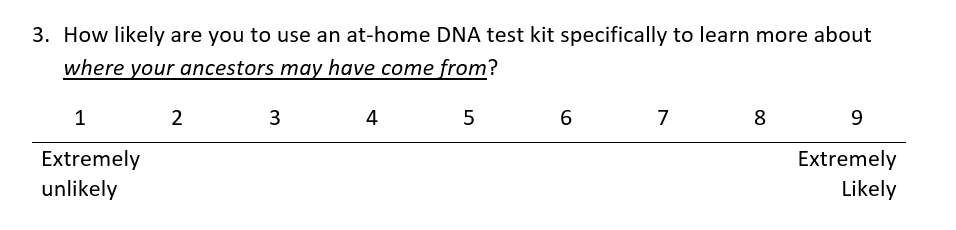


| **KINSHIP INFORMATION** |
| --- |

| **Educational Component: DTC-GT for Relatives & Family Tree Information** |
| --- |
| **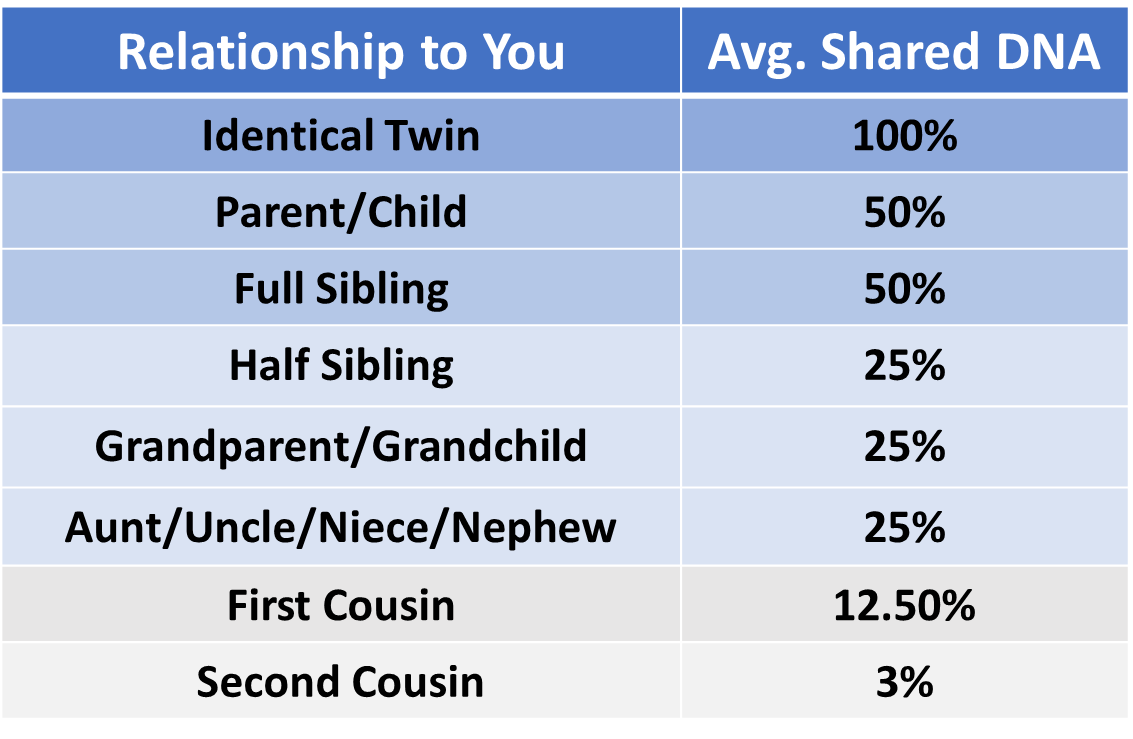Identifying Biological Relatives: How It Works**   - We share DNA with our relatives. Looking at the amount we share can tell us information about the relationship.   **Locating Biological Relatives: How It Works**  [A stock image shows ‘Jane’ with an example of a DTC-GT ‘family tree’ portal reflecting pictures and contact information of individuals to whom Jane is biologically related., along with a descriptor of relatedness (e.g., 2^nd^ – 4^th^ cousin; grandmother/granddaughter/half sister).]  *Key Points:*   - How law enforcement agencies may use genetic data (to investigate murder, assault, etc.) - Examples of how kit data have been used in the past, including that data can identify you if you’ve committed a crime, or to identify your relatives if they’ve been involved in one - Options to grant or refuse permission for access/use |

Q10. Is there anything new or surprising that you didn’t know before?

Q11. How much does the literal biological relatedness of your kin matter to you? In other words, how much does it matter whether your family members are related to you by blood or not?

Q12. How would you feel about learning about previously unknown relatives?

1. How would you feel if a previously unknown relative reached out to you?
2. How would you feel about reaching out to them?

Q13. Regardless of how certain you might be about who your blood relatives are, let’s imagine you got a result that suggested that someone in your family who you *thought* was a blood relative actually is *not* related to you by blood. What would that mean to you?

Q14. Now that you’ve had a chance to think through what different kinds of results about your kin might mean, how likely would you be to pursue testing *specifically to learn more about* ***who you are biologically related to***?


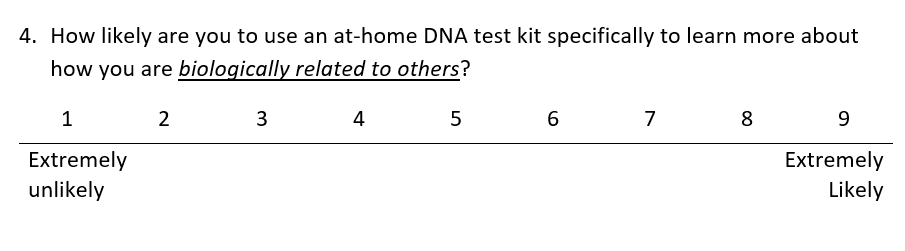


| **LAW ENFORCEMENT USE** |
| --- |
| **Educational Component: Use of DTC-GT Data for Law Enforcement Purposes** |
| **Law Enforcement and At-Home DNA Testing Companies**   - Some companies allow police to use their “Family Finder” services in a way similar to regular customers. - Those that do usually provide a choice to customers about whether they want their results to be visible to police. - Most companies do not allow police to use their services.   **Locating Biological Relatives: How It Works**  [*Jane’s family tree is presented alongside an icon of an unidentified crime suspect. A distant biological relative of Jane’s—with name, degree of relation, and contact information—is indicated as a potential match to a biological sample collected at a crime scene*.]  *Key Points:*   - How law enforcement agencies may use genetic data (to investigate murder, assault, etc.) - Examples of how kit data have been used in the past, including that data can identify you if you’ve committed a crime, or to identify your relatives if they’ve been involved in one - Options to grant or refuse permission for access/use |

Q15. Is there anything new or surprising that you didn’t know before?

Q16. Let’s say just for the sake of discussion that you have decided to have testing done. As part of setting up an account, you are asked whether you are willing to allow law enforcement access to your information.

1. Let’s brainstorm some of the reasons why someone might *want* to allow law enforcement access to their information.
2. Let’s think about some of the reasons why someone might *not* want to allow law enforcement access to their information.
3. Given all the pros and cons we have discussed, how likely are you to say yes to allowing information about your family relationships to be available to law enforcement?


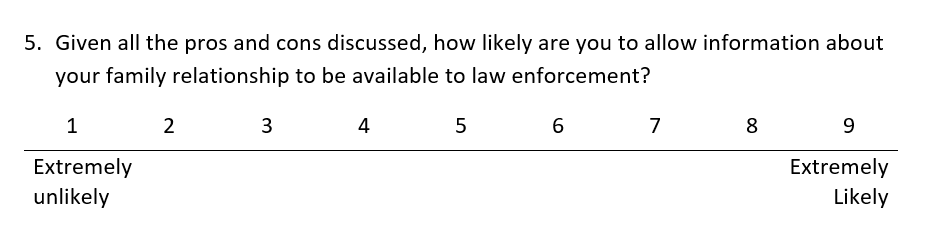


| **CONCLUDING THOUGHTS** |
| --- |

Q17. We have heard a lot of different viewpoints regarding at-home testing kits. Based on everything you’ve heard, I’d like you to rate your likelihood of having this kind of testing done one more time.


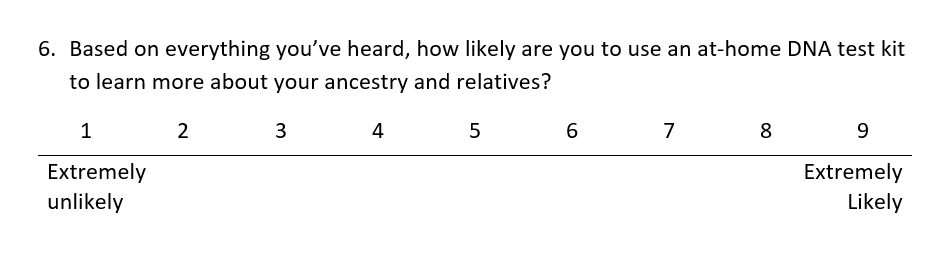


Q18. Of all the issues we've discussed today, what do you think are the most important things that people should consider when deciding whether or not to have this type of testing done?
