# Supplementary material for: Direct-to-consumer genetic testing: Prospective users’ attitudes toward information about ancestry and biological relationships
Source: PLoS One. 2021 Nov 29;16(11):e0260340. doi: 10.1371/journal.pone.0260340 (PMC8629298; doi:10.1371/journal.pone.0260340)
Supplement: S3 Appendix — (DOCX) [file pone.0260340.s003.docx]

**S3 Appendix. Participant Worksheet**

1. How likely are you to use an at-home DNA test kit to learn more about your ancestry and relatives?

| 1 | 2 | 3 | 4 | 5 | 6 | 7 | 8 | 9 |
| --- | --- | --- | --- | --- | --- | --- | --- | --- |
| Extremely unlikely | | |  | | | Extremely Likely | | |

2. How likely are you to use an at-home DNA test kit to learn more about your ancestry and relatives?

| 1 | 2 | 3 | 4 | 5 | 6 | 7 | 8 | 9 |
| --- | --- | --- | --- | --- | --- | --- | --- | --- |
| Extremely unlikely | | |  | | | Extremely Likely | | |

3. How likely are you to use an at-home DNA test kit specifically to learn more about *where your ancestors may have come from*?

| 1 | 2 | 3 | 4 | 5 | 6 | 7 | 8 | 9 |
| --- | --- | --- | --- | --- | --- | --- | --- | --- |
| Extremely unlikely | | |  | | | Extremely Likely | | |

4. How likely are you to use an at-home DNA test kit specifically to learn more about how you are *biologically related to others*?

| 1 | 2 | 3 | 4 | 5 | 6 | 7 | 8 | 9 |
| --- | --- | --- | --- | --- | --- | --- | --- | --- |
| Extremely unlikely | | |  | | | Extremely Likely | | |

5. Given all the pros and cons discussed, how likely are you to allow information about your family relationship to be available to law enforcement?

| 1 | 2 | 3 | 4 | 5 | 6 | 7 | 8 | 9 |
| --- | --- | --- | --- | --- | --- | --- | --- | --- |
| Extremely unlikely | | |  | | | Extremely Likely | | |

6. Based on everything you’ve heard, how likely are you to use an at-home DNA test kit to learn more about your ancestry and relatives?

| 1 | 2 | 3 | 4 | 5 | 6 | 7 | 8 | 9 |
| --- | --- | --- | --- | --- | --- | --- | --- | --- |
| Extremely unlikely | | |  | | | Extremely Likely | | |
